# Comparison of two techniques for toric intraocular lens implantation: hydroimplantation versus ophthalmic viscosurgical devices

**DOI:** 10.1186/s12886-018-0758-6

**Published:** 2018-04-24

**Authors:** Yueqin Chen, Qian Cao, Chunyan Xue, Zhenping Huang

**Affiliations:** Department of Ophthalmology, Jinling Hospital, School of Medicine, Nanjing University, 305 East Zhongshan Road, Nanjing, People’s Republic of China

**Keywords:** Endothelial cell density, Hydroimplantation, Intraocular pressure, Ophthalmic viscosurgical device, Toric intraocular lens

## Abstract

**Background:**

To compare the results between hydroimplantation of a single-piece, acrylic foldable toric intraocular lens (IOLs) and conventional implantation using an ophthalmic viscosurgical device (OVD).

**Methods:**

In this study, 60 eyes with cataract and preexisting regular corneal astigmatism of 1.0 to 3.0 diopters (D) underwent the implantation of the AcrySof toric IOLs (Alcon Laboratories, Inc.). The patients were randomly assigned to a conventional implantation technique with an OVD or a hydroimplantation technique. Comparison of preoperative and postoperative parameters was performed using paired Student *t* tests, and independent Student *t* test was used to compare between the two groups.

**Results:**

Three months postoperatively, the mean subjective astigmatism was 0.45 D ± 0.24 (SD) in the OVD group and 0.49 ± 0.29 D in the hydroimplantation group (*P* = 0.492). The mean endothelial cell density (ECD) loss was 7.54% ± 0.82% and 7.32% ± 0.59%, respectively (*P* = 0.117). The mean absolute IOL rotation was 4.77 ± 2.32 degrees and 4.70 ± 1.95 degrees, respectively (*P* = 0.334). The mean time for IOL implantation was 71.50 ± 8.10 s and 37.60 ± 3.90 s, respectively (*P* < 0.001). Two hours, 1 day, 1 week, 1 month, and 3 months postoperatively, there was no significant difference in IOP between the two groups (*P* > 0.05), although IOP two hours postoperatively seemed to be a little higher in the OVD group.

**Conclusions:**

Compared with the use of OVDs for toric IOLs implantation, the hydroimplantation technique provided advantages of increased efficiency, reduced surgical time and cost, and no concerns of OVD-induced elevated IOP.

**Trial registration:**

Current Controlled Trials ISRCTN55696872, Retrospectively registered (Date of registration: 25 March 2018).

## Background

It is estimated that 25% to 40% of cataract patients have astigmatism more than 1.0 diopters (D) [1, 2]. With the increasing demands for good refractive outcomes after cataract surgery, toric intraocular lenses (IOLs) have been widely used to correct preoperative corneal astigmatism during surgery. Many studies have shown that the toric IOL implantation offers a predictable and stable procedure for the correction of preexisting corneal astigmatism, and induces a low amount of higher-order aberration [1, 3–6]. The key to achieve a good reduction of astigmatism is the precise alignment of the toric IOL axis on the planned meridian. In the standard procedure, after the injection of the toric IOL, the ophthalmic viscosurgical device (OVD) should be aspirated from the anterior chamber, including from behind the lens. This procedure is crucial for the toric IOL axis being finally positioned on the planned meridian. And it could be especially difficult when one haptic of the toric IOL is located adjacent to the main incision.

Hydroimplantation,implantation of a foldable IOL without an OVD, was introduced by Tak [7], and adopted and modified by other surgeons later [8–12]. To our knowledge, there has not been any study about comparison between hydroimplantation and conventional technique for the implantation of toric IOLs. However, the hydroimplantation technique can make the toric IOL implantation much easier by skipping two steps of rotation, and the toric IOL could be rotated to its final meridian directly.

The aim of this study was to evaluate and compare the clinical results between the OVD group and the hydroimplantation group for the implantation of a single-piece, acrylic foldable toric IOL.

## Methods

### Patient population

This randomized prospective study comprised 60 eyes with cataract and preexisting regular corneal astigmatism between 1.0 and 3.0 diopters (D) who had implantation of an AcrySof toric IOL (Alcon Laboratories, Inc.). The surgeries were performed between Jan 2012 and May 2014 by one experienced surgeon (Dr. Chunyan Xue) at the Refractive Center, Department of Ophthalmology, Jinling Hospital, Nanjing, China. The eyes were randomized prospectively into two groups (the OVD group and the hydroimplantation group) according to the statistical random table. The study protocol was approved by the Ethical Committee of Jinling Hospital. All patients were fully informed of the details and possible risks of the procedure, and written informed consents were obtained from all the patients. Described research adhered to the tenets of the Declaration of Helsinki.

Inclusion criteria were age-related cataract with preoperative corneal astigmatism between 1.0 and 3.0 D and nucleus sclerosis up to grade 3. Preoperative corneal astigmatism was measured by keratometry (IOLMaster, Zeiss Humphrey, Carl Zeiss Meditec, Inc., Dublin, CA 94568). Exclusion criteria were history of previous ocular surgery, pupil size less than 7.5 mm after dilatation, anterior chamber less than 2.25 mm, compromised endothelial cell function, corneal disorder, complicated cataract, glaucoma, pseudoexfoliation, severe myopia, and diabetic retinopathy.

### Preoperative assessment

Preoperative evaluations included subjective refraction, uncorrected visual acuity (UCVA), best corrected visual acuity (BCVA) (Snellen or “E” chart), keratometry (IOLMaster, Zeiss Humphrey, Carl Zeiss Meditec, Inc., Dublin, CA 94568), corneal topography (Humphrey ATLAS Corneal Topography System, Carl Zeiss Meditec, Inc., Dublin, CA 94568), intraocular pressure (IOP) (Computerized Tonometer CT-80A, Topcon Corp. Tokyo, Japan), endothelial cell density (ECD) (SP-3000P, Topcon Corp. Tokyo, Japan), slitlamp examination (PS-11E, Topcon Corp. Tokyo, Japan), and indirect fundus examination.

### Intraocular Lens

The AcrySof toric IOL SN6ATT (Alcon Laboratories, Inc., Fort Worth, TX, USA) has open-loop modified L-haptics with 3 reference dots on each side that mark the axis of the cylinder on its posterior surface. The IOL can filter ultraviolet and blue light and is a single-piece IOL made of hydrophobic acrylic material with a refractive index of 1.55, a 6.0-mm optic diameter, and a 13.0-mm overall length. The spherical IOL power was calculated using the IOLMaster (Carl Zeiss Meditec AG) and the SRK/T formula. The cylinder power and the alignment axis were calculated using the Acrysof toric calculator available from Alcon Laboratories (www.acrysoftoriccalculator.com). The main incision was created at 135° and the surgical induced astigmatism was set as 0.5 D at 45°according to the data of previous operations of the same surgeon.

### Surgical technique

Preoperatively, limbal reference marks of the astigmatic axis were made under a slit lamp directly [13]. The patients were sitting at the slit lamp and instructed to look at a distant target at head height with the fellow eye. The slit light was centered on the apex of the cornea and turned in the steep astigmatic meridian in the orthograde position using the rotator switch of the slit light. Then, two points of the astigmatic meridian at the limbus were marked with a marking pen. All of the markings were done by the surgeon.

5% Tropicamide with 5% phenylephrine eye drops were used for preoperative pupillary dilation. The surgery was performed using the Infiniti Vision System (Alcon Laboratories, Inc.). In both groups, after topical anesthesia with oxybuprocaine hydrochloride 0.4% eye drops, a 2-step superior clear corneal incision with an approximate 2.0 mm chord length was created with a 2.2 mm Intrepid dual-bevel slit knife (Alcon Laboratories, Inc.), and a side port was created at 2 o’clock at the limbus using a paracentesis knife (Alcon Laboratories, Inc.). After the anterior chamber was filled with DiscoVisc OVD (Alcon Laboratories, Inc.), a continuous curvilinear capsulorhexis measuring 5.0 to 5.5 mm in diameter was generated using capsular forceps. Hydrodissection and hydrodelineation were performed. Following in-the-bag phacoemulsification using the quick-chop technique, the rest of the cortex was removed by irrigation/aspiration using the Intrepid silicone-sleeved coaxial system (Alcon Laboratories, Inc.).

In the OVD group, the anterior chamber and the capsular bag was filled with DiscoVisc (Alcon Laboratories, Inc.). The toric IOL was placed in the cartridge which was lubricated with DiscoVisc. The IOL was then implanted into the capsular bag using a D cartridge mounted on a Monarch II injector (Alcon Laboratories, Inc.) by the surgeon. The injector was held with the left hand and rotated for advancement of the IOL with the other hand. After the IOL was injected into the capsular bag, gross alignment was achieved by rotating the IOL clockwise until it was placed 20–30 degrees short of the planned axis. After the DiscoVisc was thoroughly removed from the eye and from behind the IOL, the IOL was rotated to its final position by exactly aligning the reference marks on the toric IOL with the alignment axis marks. A final check was made after the anterior chamber was filled and the incisions were hydrated.

**Fig. 1:**
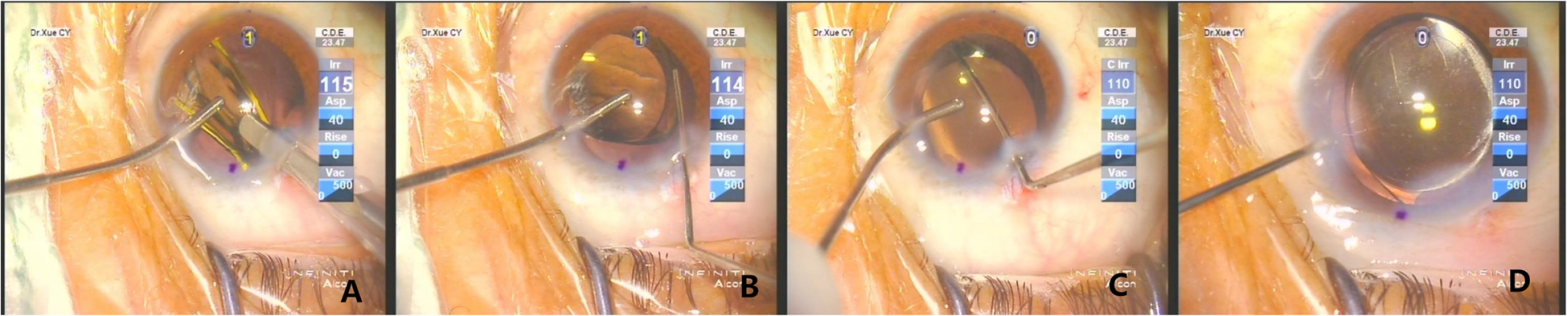
Implantation of a toric IOL. **a** The leading haptic is inserted into the capsular bag with the help of the irrigation cannula tip. **b** The trailing haptic is dialed and inserted into the capsular bag with the help of the small tip of a cyclodialysis spatula. **c** The toric IOL is rotated to its planned position directly. **d** A final check that the axis of the IOL is on the planned meridian and the incisions are hydrated

In the hydroimplantation group, following the removal of any residual cortical material, balanced salt solution (BSS) (Alcon Laboratories, Inc.) was used to maintain the anterior chamber shape instead of the DiscoVisc. To implant the IOL, the irrigation cannula was inserted into the anterior chamber through the left side port. The IOL was gradually injected into the eye through the tip of the cartridge with the assistance of an assistant who screwed the end of the injector, and the tip of the irrigation cannula could be used to guide the IOL into the capsular bag. With the help of the irrigation cannula tip and the small tip of a cyclodialysis spatula, the IOL was rotated to its final position by exact alignment of the reference marks on the toric IOL with the alignment axis marks. Finally, the incisions were hydrated (Fig. 1).

Tobramycin-dexamethasone ointment was applied to the eye at the end of the surgery. Postoperatively, tobramycin and dexamethasone eye drops were applied topically every hour. The eye drops were tapered after 1 week and then discontinued after 2 weeks.

### Postoperative assessment

Postoperative IOP were performed at 2 h, 1 day, 1 week, 1 month, and 3 months after surgery. Postoperative UDVA, ECD, refractive astigmatism (keratometry: IOLMaster, Zeiss Humphrey, Carl Zeiss Meditec, Inc., Dublin, CA 94568), IOL rotation were also evaluated after surgery. The time for the IOL implantation was recorded during the surgery. Rotation of the toric IOL was evaluated by a slit lamp: A thin slit beam was rotated to the axis markings of the IOL, and then the orientation of the IOL was estimated in 1-degree steps [14]. Astigmatism vector analysis was performed using Thibos and Horner’s power vector notation [15].

### Statistical analysis

The results were analyzed using SPSS software (version 17.0, SPSS, Inc.). Data were presented as Mean ± SD. Visual acuity was converted to logarithm of the minimum angle of resolution (logMAR) before statistical analysis. For quantitative variables, independent Student *t* test was used to compare between the two groups. Comparison of preoperative and postoperative parameters was performed using paired Student *t* tests. A normal distribution check (Kolmogorov-Smirnov test) was performed to validate the use of a Student *t* test. Qualitative variables were analyzed using the *Chi-square* test. A *P* value less than 0.05 was considered statistically significant.

## Results

Sixty eyes were included in this study. All the eyes had successful operations with total surgical time less than 20 min, and no eye had posterior capsule rupture or other complications during the surgery. Table 1 shows the demographic data. No statistically significant differences in age, sex, UCVA, BCVA, IOP, ECD, or refractive astigmatism were observed between the two groups before surgery (*P* > 0.05).

**Table 1:** Demographic data at baseline

Variables	OVD group	Hydroimplantation group	*P* value
Number	30	30	
Gender (M/F)	10/15	11/17	0.480
Age (years)	69.53 ± 10.33	67.53 ± 9.14	0.513
UCVA (logMAR)	0.93 ± 0.25	0.81 ± 0.14	0.536
BCVA (logMAR)	0.67 ± 0.24	0.68 ± 0.22	0.483
ECD (cells/mm^2^)	2418.66 ± 322.97	2529.71 ± 317.50	0.939
IOP (mmHg)	16.15 ± 2.48	15.29 ± 2.31	0.803
Refractive astigmatism (D)	2.29 ± 0.44	2.10 ± 0.42	0.804
Toric IOL model, n (%)			
T3	9 (30.0)	11 (36.7)	
T4	9 (30.0)	8 (26.7)	
T5	11 (36.7)	10 (33.3)	
T6	1 (3.3)	1 (3.3)	

*OVD* ophthalmic viscosurgical device, *UCVA* uncorrected visual acuity, *BCVA* best-corrected visual acuity, *ECD* endothelial cell density, *IOP* intraocular pressure, *D* diopters

### Visual acuity

After surgery, UCVA improved significantly in all the patients (*P* < 0.001). Three months postoperatively, the mean UCVA was 0.19 ± 0.11 logMAR (range 0.00 to 0.40 logMAR) in the OVD group and 0.19 ± 0.12 logMAR (range 0.00 to 0.40 logMAR) in the hydroimplantation group. There was no statistically significant difference in UCVA between the two groups (*P* = 0.550).

### Refractive outcomes

**Fig. 2:**
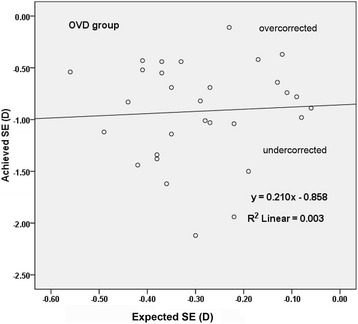
Achieved SE correction versus expected SE correction 3 months postoperatively in OVD group. (*SE* spherical equivalent, *OVD* ophthalmic viscosurgical device)

**Fig. 3:**
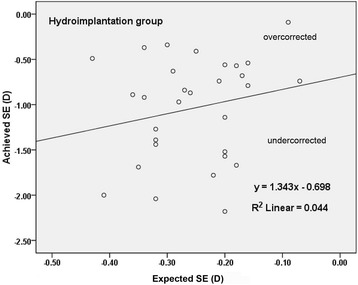
Achieved SE correction versus expected SE correction 3 months postoperatively in hydroimplantation group. (*SE* spherical equivalent)

There was a significant reduction of refractive astigmatism in the two groups after surgery (*P* < 0.001). Three months postoperatively, the mean refractive astigmatism was 0.45 ± 0.24 D (range 0.14 to 1.01 D) in the OVD group and 0.49 ± 0.29 D (range 0.12 to 1.28 D) in the hydroimplantation group. There was no statistically significant difference in refractive astigmatism between the two groups (*P* = 0.492). Figures 2 and 3 show the linear regression about the expected vs achieved spherical equivalent (SE) 3 months postoperatively in the 2 groups, and 28 eyes (93.3%) in the OVD group and 27 eyes (90.0%) in the hydroimplantation group were within ±1.50 D of target refraction.

**Fig. 4:**
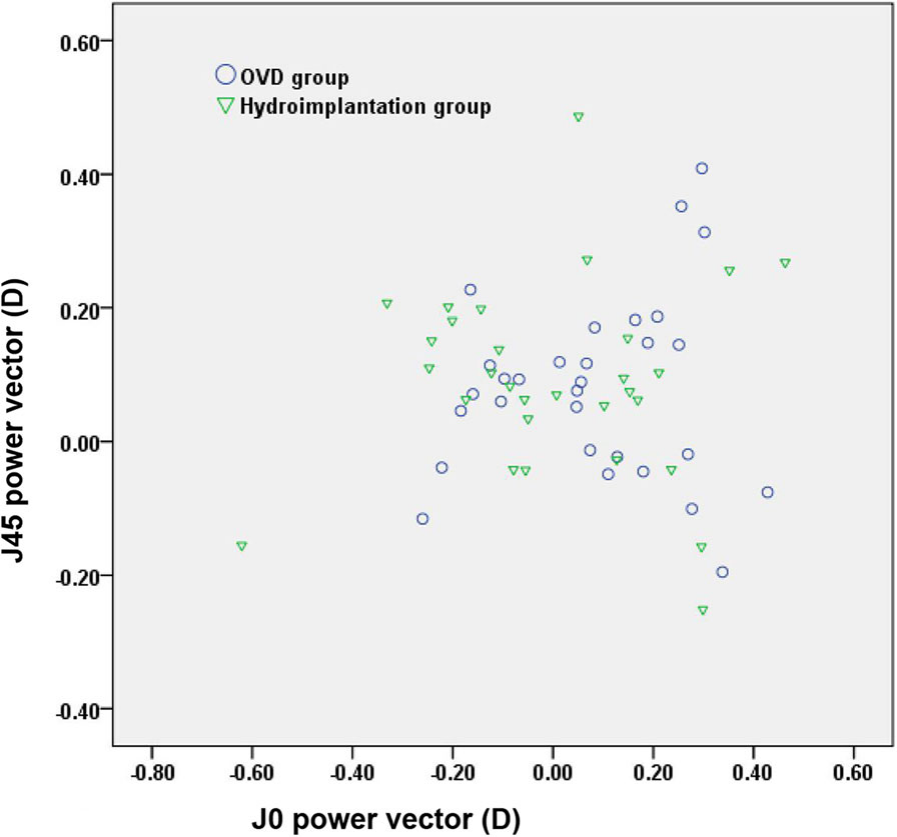
Astigmatism vectors. The x-axes represent the J0 power vector (D). The y-axes represent the J45 power vector (D). (*OVD* ophthalmic viscosurgical device, *J0* Jackson cross-cylinder, axes at 0 degree and 90 degree, *J45* Jackson cross-cylinder, axes at 45 degree and 135 degree)

Figure 4 shows the postoperative astigmatism power vector in both groups. Three months postoperatively, the mean Jackson cross-cylinder at J0 and J45 was 0.08 ± 0.19 D (range − 0.26 to 0.43 D) and 0.08 ± 0.14 D (range − 0.20 to 0.41 D) respectively in the OVD group, and 0.00 ± 0.23 D (range − 0.62 to 0.46 D) and 0.09 ± 0.15 D (range − 0.25 to 0.49 D) respectively in the hydroimplantation group. There was no statistically significant difference in J0 (*P* = 0.287) or J45 (*P* = 0.992) between the two groups.

### Endothelial cell density

Three months after surgery, the mean ECD was 2235.50 ± 294.33 cells/mm^2^ (range 1723.4 to 2765.8 cells/mm^2^) in the OVD group and 2343.59 ± 287.43 cells/mm^2^ (range 1712.6 to 2699.4 cells/mm^2^) in the hydroimplantation group. The mean ECD loss was 7.54% ± 0.82% and 7.32% ± 0.59%, respectively (*P* = 0.117).

### Intraocular pressure

Table 2 shows the IOP in 2 h, 1 week, 1 month, and 3 months after surgery. There was no significant difference in IOP between the two groups during the follow up (*P* > 0.05), although IOP two hours postoperatively seemed to be a little higher in the OVD group.

**Table 2:** Mean IOP change (mmHg) postoperatively

Time	OVD group	Hydroimplantation group	*P* value
2 h	16.25 ± 3.38	14.79 ± 3.02	0.830
1 day	14.98 ± 3.52	14.13 ± 2.83	0.119
1 week	13.45 ± 2.56	13.56 ± 2.35	0.609
1 month	14.15 ± 1.72	13.47 ± 1.78	0.715
3 months	14.70 ± 1.99	14.08 ± 1.93	0.981

*IOP* intraocular pressure, *OVD* ophthalmic viscosurgical device

### Intraocular lens rotation

**Fig. 5:**
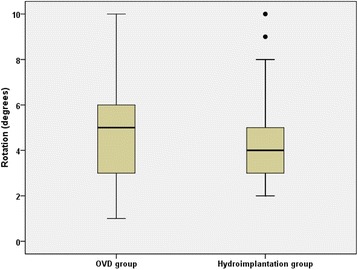
Absolute axis rotation between the two groups three months postoperatively. (OVD = ophthalmic viscosurgical device)

Three months postoperatively, the mean absolute IOL rotation relative to the intended meridian was 4.77 ± 2.32 degrees (range 1 to 10 degrees) in the OVD group and 4.70 ± 1.95 degrees (range 2 to 10 degrees) in the hydroimplantation group. There was no statistically significant difference in IOL rotation between the two groups (*P* = 0.334) (Fig. 5).

### Time for intraocular lens implantation

The time for the IOL implantation was calculated from the end of complete removal of the cortex to the beginning of incision hydrated. The mean time for the toric IOL implantation was 71.50 ± 8.10 s in the OVD group and 37.60 ± 3.90 s in the hydroimplantation group. The time for the IOL implantation was much less in the hydroimplantation group (*P* < 0.001).

## Discussion

There are many studies about foldable IOLs implantation using anterior chamber infusion, but no comparison study about toric IOLs implantation. In this study, we find that the advantages of the hydroimplantation over OVDs are increased efficiency and reduced cost [16]. During the IOL implantation, the IOL can be rotated to its final position directly, skipping two steps of rotation using hydroimplantation compared with OVDs. Therefore, the hydroimplantation technique leads to deceased surgery time and increased efficiency, which is important in high-volume surgical centers. In our study, the mean time for the toric IOL implantation was 71.50 s in the OVD group and 37.60 s in the hydroimplantation group. The time for the IOL implantation was much less in the hydroimplantation group. Additionally, if smaller sized OVD syringes become available in the future, the hydroimplantation technique may reduce costs. And the manufacturer may produce OVD with smaller size and less cost.

UCVA and refractive astigmatism are important parameters of success for cataract patients with preoperative corneal astigmatism undergone implantation of toric IOLs. Three months after surgery, UDVA and refractive astigmatism were improved in both groups, and there were no significant differences between the two groups in UDVA or in refractive astigmatism. Three months postoperatively, the mean absolute IOL rotation relative to the intended meridian was 4.77 degrees with a range from 1 to 10 degrees in the OVD group and 4.70 degrees with a range from 2 to 10 degrees in the hydroimplantation group. The rotation of the IOL was similar between the two groups. Complete IOL fixation on the posterior capsule is another advantage of hydroimplantation [10], which is more important for AcySof toric IOL. The stability of AcySof toric IOL in our study is inferior to the findings by Waltz, Hirnschall, and Lee et al. with the mean rotation from 2.2 to 4.1 degrees [17–20]. This result may be related to the evaluation method, preoperative marking, axis placement during surgery, and complete removal of the OVD or using hydroimplantation method.

In this study, BSS was used with a modification of Michael Blumenthal’s AC chamber maintainer to maintain the anterior chamber and expand the bag [21]. Nonetheless, this study was carried out on normal corneas and foldable acrylic IOLs. Besides, the technique has limitations in compromised anterior chamber stability compared with OVDs. Therefore, we must be careful to abandon OVDs only when we can safely expand the bag, and it should be evaluated whether it could be applicable for diseased corneas and other IOLs. We do not recommend this technique for novice ophthalmologists. Mackool suggests using this technique in normal cases and to keep the OVD implantation technique for IOLs that open abruptly and complicated cases such as posterior capsular rupture, torn anterior capsulorhexis, and floppy-iris syndrome [9].

In the hydroimplantation method, the irrigation cannula was inserted into the anterior chamber through the left side port with the surgeon’s left hand and the injector held with the other hand. It was not a 2-handed technique when the IOL was implanted using a manual Monarch injector. The IOL was injected into the eye with the aid of an assistant who screwed the end of the injector. If a motorized IOL injector was used, the injection procedure with hydroimplantation would be a 2-handed technique [22]. Furthermore, this procedure would be easy and safe when the eye was stabilized by the irrigation cannula in the side port.

As Lee reported that using BSS for maintaining the anterior chamber without OVD during IOL implantation did not cause a significant difference in ECD loss 3 months after surgery, our study showed the same results [11]. There was no significant difference in ECD loss between the two groups in our study. Lee revealed that use of BSS during IOL implantation resulted in reduction in postoperative IOP spike compared with the use of OVD [11]. But in our study, we did not find any difference in postoperative IOP between the two groups, although IOP two hours postoperatively seemed to be a little higher in the OVD group (16.25 mmHg in the OVD group versus 14.79 mmHg in the hydroimplantation group). This may be due to the small sample in our study or incomplete removal of the OVD in Lee’s study. Of note, previously vitrectomized eyes were included in Lee’s study which were absent in our study. The study by Studeny et al. found no influence of the implantation technique (OVD or hydroimplantation) on postoperative IOP changes, which was comparable to our study [9]. However, larger studies may be needed to evaluate the influence of the two techniques on postoperative IOP, especially at different time point on the first postoperative day.

## Conclusion

In conclusion, the hydroimplantation technique provides comparable outcomes to the conventional technique using OVDs for the implantation of toric IOLs, however, it has the advantages of increased efficiency, reduced surgical time and cost (if smaller size of OVD is available), and no concerns of OVD-induced elevated IOP. Furthermore, this technique is useful for the alignment of toric IOLs during the surgery, and the stability of the toric IOLs after surgery. However, the hydroimplantation cannot completely replace the OVD technique since this technique has advantages in complex cases. Importantly, this technique is only recommended to experienced surgeon, for novice ophthalmologists may cause some tough intraoperative complications. Thus, hydroimplantation technique may be an alternative method for the implantation of a single-piece, acrylic foldable toric IOL in some cases.

## Abbreviations

BBS: Balanced salt solution
BCVA: Best-corrected visual acuity
ECD: Endothelial cell density
IOLs: Intraocular lens
IOP: Intraocular pressure
LogMAR: Logarithm of the minimum angle of resolution
OVD: Ophthalmic viscosurgical device
SE: Spherical equivalent
UCVA: Uncorrected visual acuity
